# Direct Pyrolysis of a Manganese‐Triazolate Metal–Organic Framework into Air‐Stable Manganese Nitride Nanoparticles

**DOI:** 10.1002/advs.202003212

**Published:** 2021-01-04

**Authors:** Yating Hu, Changjian Li, Shibo Xi, Zeyu Deng, Ximeng Liu, Anthony K. Cheetham, John Wang

**Affiliations:** ^1^ Department of Materials Science and Engineering National University of Singapore 9 Engineering Drive 1 Singapore 117574 Singapore; ^2^ Function Hub Hong Kong University of Science and Technology (Guangzhou) S&T Building, Nansha IT Park Guangzhou 511458 China; ^3^ Institute of Chemical and Engineering Sciences Agency for Science, Technology and Research (A*STAR) 1 Pesek Road, Jurong Island Singapore 627833 Singapore; ^4^ Materials Research Laboratory University of California Santa Barbara CA 93106 USA

**Keywords:** air‐stable nanoparticles, electrocatalysis, metal–organic frameworks, pyrolysis, transition‐metal nitrides

## Abstract

Although metal–organic frameworks (MOFs) are being widely used to derive functional nanomaterials through pyrolysis, the actual mechanisms involved remain unclear. In the limited studies to date, elemental metallic species are found to be the initial products, which limits the variety of MOF‐derived nanomaterials. Here, the pyrolysis of a manganese triazolate MOF is examined carefully in terms of phase transformation, reaction pathways, and morphology evolution in different conditions. Surprisingly, the formation of metal is not detected when manganese triazolate is pyrolyzed in an oxygen‐free environment. Instead, a direct transformation into nanoparticles of manganese nitride, Mn_2_N*_x_* embedded in N‐doped graphitic carbon took place. The electrically conductive Mn_2_N*_x_* nanoparticles show much better air stability than bulk samples and exhibit promising electrocatalytic performance for the oxygen reduction reaction. The findings on pyrolysis mechanisms expand the potential of MOF as a precursor to derive more functional nanomaterials.

## Graphical abstract

The pyrolysis of a manganese triazolate metal–organic framework (MOF) is examined in detail to reveal the mechanism in deriving MOF into functional nanomaterials. Surprisingly, a direct transformation of MOF into Mn_2_N*_x_* nanoparticles instead to metal is observed. Moreover, the Mn_2_N*_x_* shows much better air stability than bulk samples, due to the graphitic carbon layers in which the nanoparticles are enclosed.

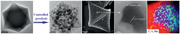

Metal–organic frameworks (MOFs) formed by linking inorganic and organic building units have emerged as versatile precursors for obtaining carbonaceous or metal‐based nanomaterials for applications in energy storage or electrocatalysis.^[^ ^1^ ^]^ During a typical MOF pyrolysis process, metallic nanoparticles, or even nanoclusters containing just a few atoms, are initially formed as the organic linkers are lost.^[^ ^2^ ^]^ For metals with a reduction potential of less than −0.27 V, metal oxide nanoparticles may become the final product, when the MOF linker contains oxygen.^[^ ^3^ ^]^ Alternatively, metal carbide nanoparticles can be formed when the MOF‐derived metallic species react with the carbon that forms from the organic linkers (e.g., at a temperature of higher than 1600 °C, MOF‐derived Co metal nanoparticles react with carbon to form Co_3_C).^[^ ^4^ ^]^ When the metallic species are removed without undergoing further reaction, porous carbons can be left behind.^[^ ^5^ ^]^ These MOF‐derived carbonaceous or metal‐based nanomaterials show great potential in energy‐related fields due to the high electrical conductivity provided by the carbon combined with the uniformly distributed active sites.^[^ ^6^ ^]^ In some cases, the uniform pores arising from the MOF precursor are used as a constraint, preventing agglomeration and coalescence of the in situ generated nanocrystals.^[^ ^7^ ^]^ Although various metals/metal oxides and porous carbons can be derived from MOFs through pyrolysis and further treatment,^[^ ^2^, ^8^ ^]^ the mechanisms involved in MOF pyrolysis remain unclear in many respects. Furthermore, to the best of our knowledge, there is no report of a one‐step reaction that transforms the MOF into a transition metal compound while making simultaneous use of both the metal ion and the organic linker in the MOF.

Transition metal nitrides (TMNs) exhibit higher electrical conductivities than the corresponding oxides and higher stabilities than the carbides during electrocatalytic reactions, such as in oxygen reduction reactions (ORR).^[^ ^9^ ^]^ Upon forming nitride, the transition metal's filled state of d‐band is narrowed and unfilled state broadened. As a result, the surface of TMN is more catalytically active than its parent metal or corresponding transition metal oxides, even more active than the benchmarking Pt group metals for reactions that involve the donation of bonding electrons.^[^ ^10^ ^]^ Generally, an effective electrocatalyst requires a high surface area and reasonable electrical conductivity. Reducing the particle size of a material to the nanoscale (i.e., smaller than 50 nm) is an effective way to increase the surface area, thereby increasing the number of accessible catalytic sites.^[^ ^11^ ^]^ Recently, sub‐micrometer‐scale transition metal nitrides have being synthesized by nitridation of oxides in ammonia, or using the —NH_2_ or —NH_4_ containing precursors such as urea.^[^ ^11^ ^]^ In this way, Co nitrides with various morphologies^[^ ^6^, ^12^ ^]^ as well as NiN_3_,^[^ ^13^ ^]^ TiN,^[^ ^14^ ^]^ and VN^[^ ^15^ ^]^ have been synthesized. However, the reported ones are either relatively large in size (sub‐micrometer scale rather than nano) or lack in uniformity of size and morphology. The synthetic methods are mainly limited to the utilization of ammonia, which poses safety and environmental issues. In addition, nanoscale TMNs with higher specific surface area are difficult to obtain and stabilize as they are easily oxidized, either during their synthesis or storage.^[^ ^16^ ^]^ Review articles have pointed out that a better preparation method for nanoscale TMNs, especially those to be used in electrocatalysis, is urgently needed.^[^ ^1^, ^11^ ^]^

Herein, we studied the pyrolysis of a manganese 1,2,3‐triazolate MOF Mn(C_2_N_3_H_2_)_2_,^[^ ^17^ ^]^ under several different conditions. Interestingly, one of them leads to the formation of air‐stable manganese nitride nanoparticles, ≈30 nm in diameter, enclosed in graphitic carbon layers. The reaction mechanism has been studied by probing the phase transitions, morphology evolution, and chemical reactions involved during the pyrolysis. The manganese nitride nanoparticles show superior stability in air compared to bulk samples, mainly due to the existence of the graphitic carbon layers in which the nitride nanoparticles are enclosed and the appropriate size match between the two. The high electrical conductivity and stability of the nitride nanocrystals uniformly distributed on a carbon monolith results in better ORR electrocatalytic activity than that of manganese oxide nanomaterials.

The pyrolysis of a MOF is the thermal decomposition process involving bond breaking and transformation of the organic linker into smaller organic molecules and carbon.^[^ ^2c^ ^]^ The atmosphere used during pyrolysis plays an important role in the process, as do the heating ramp rate, holding temperature, and duration. When a manganese MOF with an oxygen‐containing organic linker is pyrolyzed, a manganese oxide is usually the final product due to Mn's high affinity toward oxygen.^[^ ^8^, ^18^ ^]^ For this reason, the oxygen‐free/nitrogen‐containing manganese triazolate (MOF MET‐2) was chosen as the precursor to obtain manganese nitride.^[^ ^17^ ^]^ To understand the mechanism of MOF pyrolysis, various in situ and ex situ characterization tools were employed. First, thermogravimetric analysis (TGA) of the manganese triazolate in flowing N_2_ gas (Figure S1a, Supporting Information) shows a mass loss at around 500 °C when the ramp rate is 5 °C min^−1^ or higher. At higher ramp rate (e.g., 10 or 30 °C min^−1^ compared to 5 °C min^−1^), the mass loss is significantly increased. The remaining mass is less than the content of manganese in MOF, indicating a likely formation of organometallic compound. The pyrolysis/annealing of the manganese triazolate was therefore carried out in flowing N_2_ using a tube furnace (ramp from room temperature in 5 °C min^−1^ and then hold at various temperatures from 500 to 700 °C). Powder X‐ray diffraction (XRD) analysis in **Figure** **1**a revealed that crystalline Mn_2_N*_x_* (*x* = 0.86) is formed from the pyrolysis/annealing at 525 °C for 4 h, in highly purified N_2_ atmosphere. Sample obtained from pyrolysis/annealing at 525 °C for 4 h is coded as MN/NGC (MN stands for manganese nitrides and NGC stands for N‐doped graphitic carbon, which will be further discussed). Through examining the pyrolyzed products obtained from various heat treatments conducted in the tube furnace, it is found that the manganese triazolate is highly sensitive to air at higher temperatures (>400 °C). Pure Mn_2_N*_x_* can only be formed when an oxygen trap is attached to the N_2_ gas hose; this reduces the oxygen content to less than 50 ppb, compared to up to 2 ppm in the gas taken directly from the commercial N_2_ cylinder. A mixture of MnO and Mn_2_N*_x_* or highly crystalline MnO is obtained when the oxygen trap is removed or when the pyrolysis is done in air, respectively (Figure S2, Supporting Information).

**Figure 1:**
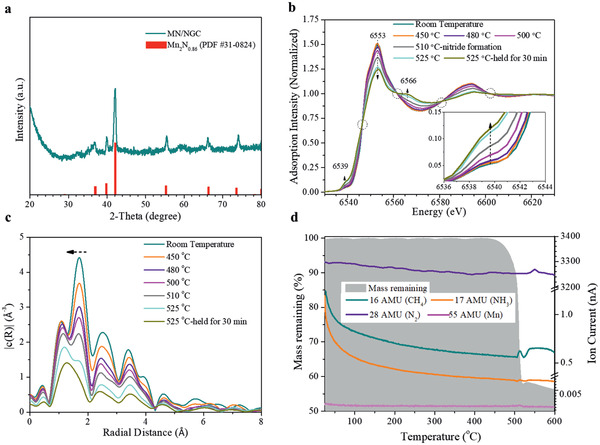
The phase transformation during the pyrolysis of manganese triazolate in pure N_2_. a) XRD of the as‐obtained pyrolysis product MN/NGC. b) In situ Mn K‐edge X‐ray absorption near‐edge structure (XANES) spectra and c) Fourier transform of the extended X‐ray absorption fine structure (EXAFS) spectra during the pyrolysis of manganese triazolate's into manganese nitride (heating up at 5 °C min^−1^ ramp in N_2_ from room temperature, then held for 30 min at 525 °C). d) Thermogravimetric analysis‐mass spectrometry (TG‐MS) results during the pyrolysis of manganese triazolate in N_2_, ramping at 5 °C min^−1^.

In situ Mn K‐edge X‐ray absorption fine structure (XAFS, including both XANES and EXAFS) spectra were measured during the pyrolysis of manganese triazolate in highly purified N_2_ gas to shed further light on the reaction mechanism. Data were collected while the manganese triazolate was heated from room temperature with a ramp rate of 5 °C min^−1^ under highly purified N_2_ gas flowing at 100 mL min^−1^. The XANES data collected at 200, 300, and 400 °C show no change (Figure S3, Supporting Information) because the manganese triazolate is thermally stable up to 400 °C (Figure S4, Supporting Information).^[^ ^17^ ^]^ Furthermore, the XANES profiles at 450 and 480 °C show negligible differences from the one at room temperature (Figure 1b). Starting from 510 °C, however, the intensity of the peak at 6539 eV increases significantly. This pre‐edge peak, which is about 10 eV below the main peak, corresponds to an electronic excitation from 1s to 3d for first‐row transition metals.^[^ ^19^ ^]^ The peak becomes more intense when the coordination environment of the metal atom is distorted from a centrosymmetric to a non‐centrosymmetric one.^[^ ^20^ ^]^ None of the manganese oxides show a peak at this energy.^[^ ^21^ ^]^ In this case, the pre‐edge peak at 6539 eV is associated with the formation of manganese nitride Mn_2_N*_x_*,^[^ ^19^ ^]^ because the symmetry at the Mn site is non‐centrosymmetric due to the presence of adjacent nitrogen vacancies (**Scheme** **1**a). The relative intensity of the pre‐edge peak compared with the main adsorption peak at 6553 eV increases when temperature is raised above 500 °C, as the manganese environment gradually changes from the centrosymmetric coordination in manganese triazolate^[^ ^17^ ^]^ to the non‐centrosymmetric environment in Mn_2_N*_x_*. The clear‐cut intersection points of the curves (isosbestic points) are evidences of the transformation between two phases (manganese triazolate to Mn_2_N_0.86_), taking place during the heat treatment. The overall peak profile, especially the post‐edge peak at 6566 eV which becomes obvious at 525 °C, matches the reported peaks for manganese in manganese nitrides.^[^ ^22^ ^]^ The peak at 6553 eV that remained throughout the pyrolysis, and the absence of a peak at 6580 eV, exclude the formation of various manganese oxides^[^ ^23^ ^]^ and metallic manganese.^[^ ^22^ ^]^ To underline this point, the in situ XANES results, especially the isosbestic points, confirm that when manganese triazolate is pyrolyzed in highly purified N_2_, metal nitride is formed directly, rather than via a manganese metal phase. This result is strikingly different from other published studies on the pyrolysis of MOFs, where the metal ions initially form metal clusters or nanoparticles.^[^ ^2^, ^3^, ^4^ ^]^ The Fourier transform of EXAFS spectra (Figure 1c) shows a decrease in the Mn—N distance during pyrolysis,^[^ ^24^ ^]^ which results from the transformation from manganese triazolate (Mn—N 2.16 Å) to Mn_2_N*_x_* (1.98 Å) (Scheme 1a).

**Scheme 1:**
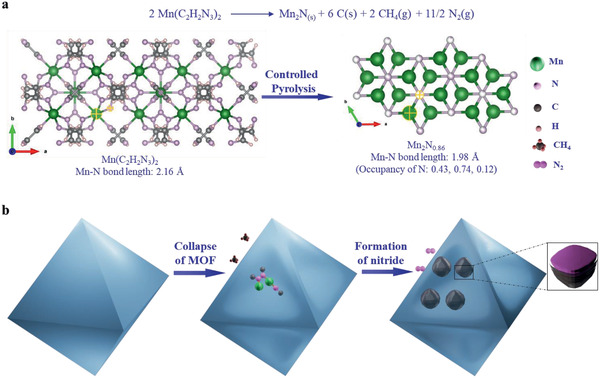
Reaction pathway and morphology change from manganese triazolate to manganese nitride nanoparticles. a) Proposed overall reaction and the crystal structures of Mn(C_2_H_2_N_3_)_2_ (0.5 unit cell) and Mn_2_N_0.86_. b) Illustration of the morphology evolution during the pyrolysis.

When conducting in situ scanning transmission electron microscopy (STEM) imaging to observe the morphology evolution during the pyrolysis of the MOF, it was found that the pyrolysis evolved quite differently in the high‐vacuum conditions of the STEM chamber (10^−7^ mbar). Both the element mapping (**Figure** **2**) and elemental content analysis based on electron energy loss spectroscopy (Figure S5, Supporting Information) show that N is gradually lost (especially at the surface) before any nitride is formed. Upon losing N, Mn^2+^ quickly scavenged the residual oxygen inside the STEM chamber,^[^ ^25^ ^]^ eventually forming manganese oxide nanoparticles on the surface of the original MOF. Energy‐dispersive X‐ray spectroscopy (EDS) element mappings indicate that manganese oxide starts to form at 500 °C, as O starts to accumulate at the edge and the signals from Mn and O overlaps (Figure 2e–h). It seems likely that, under high‐vacuum conditions, nitride could not be formed because nitrogen was lost as N_2_ or NH_3_ even before the temperature reached 400 °C, while the manganese triazolate is thermally stable at this temperature under atmospheric pressure. It has previously been reported that the decomposition temperatures of certain organic molecules are lower in high‐vacuum conditions.^[^ ^26^ ^]^ We conclude that the thermal stability of manganese triazolate is much lower in high‐vacuum conditions compared with atmospheric pressure, leading to very different pyrolysis behavior.

**Figure 2:**
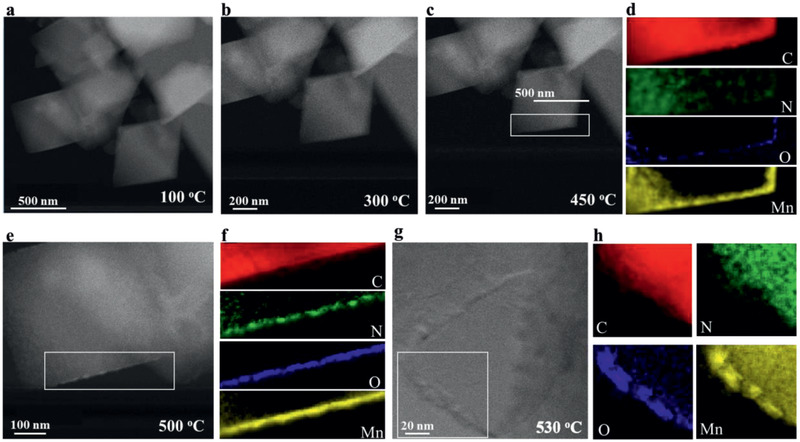
a–g) In situ STEM images when manganese triazolate was heated up from room temperature with a ramp rate of 5 °C min^−1^: at 100, 300, 450, 500, and 530 °C, respectively; d,f,h) Energy‐dispersive X‐ray spectroscopy element mappings of the boxed area in (c), (e), and (g), respectively.

Thermogravimetric analysis‐mass spectrometry (TG‐MS) was employed to further probe the pyrolysis mechanism of manganese triazolate. The same temperature ramp rate of 5 °C min^−1^ and N_2_ flow rate of 100 mL min^−1^ were used. A notable CH_4_ peak and a very weak NH_3_ peak accompanied the drop in mass at ≈500 °C, followed by an N_2_ peak at ≈550 °C (Figure 1d). Surprisingly, when conducted in air, manganese metal and triazole (C_2_H_3_N_3_) were detected (Figure S6, Supporting Information). In addition, the decomposition temperature in air is lower than that in N_2_ at various ramp rates (Figure 1b). This implies that the existence of oxygen or moisture might catalyze the decomposition of the MOF into metal and the linker molecules. The different results in N_2_ and in air indicate that the manganese triazolate's pyrolysis is significantly affected by the atmosphere. Two other metal triazolates were also synthesized: the Fe‐ and Co‐based ones (MET‐3 and MET‐4).^[^ ^17^ ^]^ However, these metal triazolates could not be converted into metal nitrides through the same heat treatment as the manganese triazolate. Instead, iron carbide (Fe_3_C) and cobalt metal were formed from MET‐3 and MET‐4, respectively (Figure S7, Supporting Information). Thus, apart from the reaction conditions, other factors such as the metal itself also affect the pyrolysis products of MOFs; such behavior is worthy of further study.

There are two possible pathways for the manganese nitride to be formed during the pyrolysis in N_2_. First, metallic Mn could be formed before reacting with N_2_ to make the manganese nitride; this would be similar to what has been reported for other MOF pyrolysis.^[^ ^2a–c^ ^]^ For bulk Mn metal, a temperature as high as 750 °C is necessary for N_2_ to oxidize Mn to manganese nitride.^[^ ^27^ ^]^ Considering the nanoscale of the metal particles formed from MOFs, a lower temperature might be sufficient. However, the formation of metal is not detected by either XRD or XAFS when the manganese triazolate is pyrolyzed in highly purified N_2_. To completely eliminate the possibility of Mn metal reacting with the N_2_ atmosphere to form manganese nitride, control runs of pyrolysis/annealing were done in Ar gas and the same Mn_2_N*_x_* nanocrystals were formed (Figure S8, Supporting Information). In addition, the formation of manganese nitride started at around 510 °C based on the in situ XAFS analysis, while N_2_ peak was detected at ≈550 °C by TG‐MS. Thus, the possibility of Mn metal reacting with released N_2_ from the MOF to form manganese nitride is rather low.

In light of the above findings on the phase transformation and identification of the products from the pyrolysis reaction, a second pathway is proposed: the direct transformation from manganese triazolate into manganese nitride. More precisely, the triazole molecule linked to Mn^2+^ decomposes at around 500 °C during the pyrolysis, beginning with N—N bond cleavage that causes ring‐opening of the triazolate, which is energetically feasible.^[^ ^28^ ^]^ Small molecules such as CH_4_ are released and the N atoms form metal nitride with Mn^2+^ at the same time as the triazole bond cleavage. N_2_ is released at a higher temperature when the N and C‐containing fragments further decompose, giving rise to the N_2_ peak at around 550 °C in Figure 1d and leaving amorphous carbon behind (see illustration in Scheme 1b). During the pyrolysis/annealing at 525 °C, N_2_ is not released but N‐doped carbon is obtained. As evidence in support of this proposal, the chemical analysis (using CHNS analyzer and inductively coupled plasma‐optical emission spectrometer) shows a lower N at% in the products when the annealing temperature is higher than 550 °C (Table S1, Supporting Information). The simplified overall reaction is proposed in Scheme 1a, based on which the Gibbs free energy (*G*_d_) for the decomposition was calculated (Table S2, Supporting Information). The negative *G*_d_ shows that the pyrolysis of the MOF into Mn_2_N (Mn_2_N*_x_* is simplified into Mn_2_N for calculation purpose), C (graphite), CH_4_, and N_2_ is energetically favored.

Chemical analysis revealed the hybrid nature of the pyrolysis product MN/NGC, confirming that it contains both Mn_2_N*_x_* nanocrystallites and N‐doped carbon (Table S1, Supporting Information). Around 57 at% of N from the MOF transferred into manganese nitride while the remaining exist as N atoms that are doped in the graphitic carbon layer or the amorphous carbon monolith. The global morphology does not change greatly upon pyrolysis (**Figure** **3** and Figure S9, Supporting Information). The manganese triazolate crystals are octahedra, 0.5 to 2 µm in size when viewed by scanning electron microscopy (Figure S9a, Supporting Information), while the 2D imaging by transmission electron microscopy (TEM) shows a hexagonal projection (Figure 3a). After pyrolysis, the octahedral monoliths are maintained (Figure 3b), but helium ion microscopy (HIM) imaging clearly shows that nanoparticles of around 30 nm are formed on the surface of the original manganese triazolate (Figure 3c,d and Figure S10, Supporting Information). The original octahedra shrank to form octahedral carbon monoliths with sunken surfaces (Figure 3b–d). When the manganese triazolate collapses, it appears that the Mn^2+^ may diffuse to the surfaces of the octahedra and react with the N atoms, as the system is N‐rich. The nanocrystallites of Mn_2_N*_x_* grow on the surface to minimize surface energy and N‐doped carbon is left behind in the bulk. High‐resolution TEM imaging and EDS element mapping (Figure 3e,f) show that the nanoparticles are fully covered by N‐doped carbon layers and reveal lattice fringes corresponding to the (111) plan of Mn_2_N_0.86_ (powder diffraction file (PDF) No. 31‐0824). The morphology evolution is summarized in Scheme 1b.

**Figure 3:**
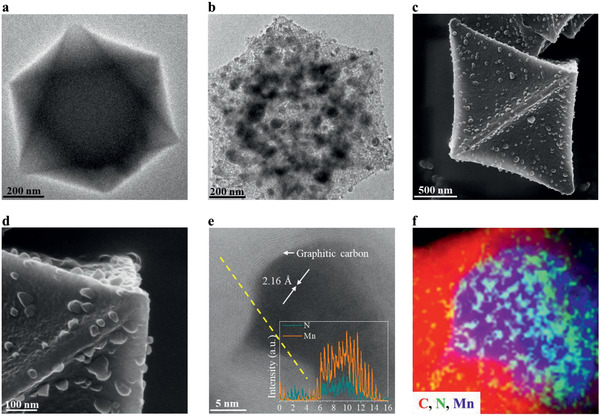
Morphology and chemical composition evolution from manganese triazolate to the manganese nitride/carbon hybrid. TEM images of the a) as‐synthesized manganese triazolate and b) MN/NGC. c,d) Helium ion microscopy (HIM) images of MN/NGC from different directions. e) High‐resolution TEM image of MN/NGC showing the lattice fringes of both Mn_2_N*_x_* and graphitic carbon layer (inset shows the Energy‐dispersive X‐ray spectroscopy line scan results of N and Mn along the yellow line). f) Elemental mapping of (e).

To assess the boundary conditions and limiting size of the manganese nitride in maintaining its stability in air, the products obtained from different pyrolysis/annealing temperatures and durations were examined. Upon increasing the annealing time from 4 to 6 h at the original temperature of 525 °C where MN/NGC was obtained, the oxygen content in the final product increased significantly (Table S1, Supporting Information). When examining its morphology using TEM (Figure S11, Supporting Information), it was found that when annealing time is extended from 4 to 6 h, the manganese nitride particles become bigger (from ≈30 nm to more than 50 nm) and the coverage of the graphitic carbon layer tends to be incomplete. In addition, some particles with lattice fringes corresponding to Mn_3_O_4_ are observed. The Mn_3_O_4_ is formed as a result of the oxidation of manganese nitride as soon as it comes into contact with air while being retrieved from the tube furnace. It is thus concluded that the graphitic‐carbon layers could not withstand annealing for 6 h, most probably due to the crystal growth of manganese nitride nanoparticles.^[^ ^29^ ^]^ The MN/NGC was kept in air to examine its air‐stability. After four weeks in air (MN/NGC‐air), the dominant crystal structure is still Mn_2_N_0.86_, while three small peaks at 34.9°, 40.5°, and 58.7° 2*θ* appeared (Figure S12, Supporting Information), which are associated with crystalline MnO (PDF No. 07‐0230). The MnO is probably formed from residual Mn^2+^ left in the bulk of the octahedron, as Mn_3_O_4_ is the product when Mn_2_N*_x_* is oxidized in air.

Pure manganese nitride is highly reactive toward oxygen and is readily oxidized.^[^ ^30^ ^]^ Indeed, when synthesizing bulk manganese nitride (Mn_3_N_2_) based on a reported method,^[^ ^31^ ^]^ it is difficult to avoid oxidation (Figure S13, Supporting Information). When higher annealing temperatures are used for the original duration of 4 h, the graphitic‐carbon layer formed at around 525 °C could not be retained at all and the manganese nitride nanoparticles were oxidized into mixture of Mn_3_O_4_ and Mn_2_N*_x_* as soon as they came in contact with air (Figures S14 and S15, Supporting Information), similar to the bulk Mn_3_N_2_ we synthesized. Thus, the size of the nitride particle is crucial in maintaining the integrity of the carbon layer so that oxidation can be prevented. In this case, 30 nm is the limiting size of the manganese nitride nanoparticles in maintaining the graphitic layer's integrity, therefore maintaining the stability of manganese nitride nanoparticle in air.

The Mn_2_N_0.86_ (111) thin film is a ferromagnetic material^[^ ^32^ ^]^ and the electronic structure of crystalline Mn_2_N_0.86_ confirms its metallic nature.^[^ ^33^ ^]^ Thus, bulk Mn_2_N_0.86_ has an electrical conductivity that is much higher than that of most metal oxides. The layered structure of the carbon in Figure 3e, with its uniform interlayer distance, suggests that it is graphitic in nature. This is confirmed by the Raman spectrum of the MN/NGC, which shows the higher intensity of the G band compared with the D band (**Figure** **4**a),^[^ ^34^ ^]^ while containing defects due to the N‐doping^[^ ^35^ ^]^ and curvature. The carbon monolith left after collapse of the MOF acts as a scaffold to hold the manganese nitride nanoparticles, maximizing their surface area. As a result, these manganese nitride nanoparticles, which are enclosed in graphitic‐carbon layers and supported by the carbon monolith, have potential interest for applications that require both catalytic activity of a transition‐metal nitride and high electrical conductivity. To explore this possibility, the ORR electrocatalytic activity of MN/NGC was tested and compared with samples that are oxidized to various extents.

**Figure 4:**
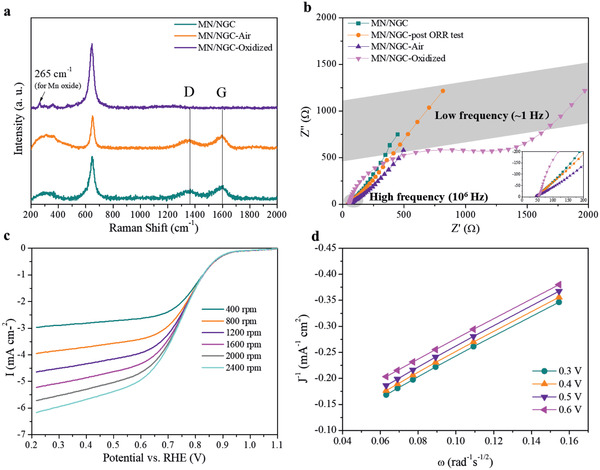
Characterizations of the manganese nitride/carbon hybrid and the oxidized samples. a) Raman spectrum and b) Nyquist plots for various samples; c) Polarization curves at different rotation speeds and d) Koutecky–Levich plots from 0.3 to 0.6 V (vs RHE) for MN/NGC.

For a better comparison between the manganese nitride and oxide in terms of electrical conductivity, a sample of MN/NGC was treated in an oven at 300 °C under air flowing for controlled oxidation (MN/NGC‐oxidized). The octahedral carbon monolith was retained, even though the nitride was fully oxidized to Mn_3_O_4_ (Figure S16, Supporting Information) and the overall carbon content is not largely affected (Table S1, Supporting Information). The Nyquist plots obtained from electrical impedance spectroscopy show that, due to complete oxidation through heat treatment in air, the electron transfer resistance of MN/NGC‐oxidized becomes much larger (Figure 4b). However, by being exposed to air for over four weeks or going through the ORR test, the electrical conductivity of the MN/NGC is hardly affected. The high electrical conductivity of the manganese nitride nanoparticles and the fact that they are uniformly distributed on the surface of the carbon monolith contributed to the diffusion‐dominant impedance profile. The ORR activities in an O_2_‐saturated alkaline aqueous solution were examined by linear sweep polarization and the onset potential at 1600 rpm was used to represent the ORR catalytic activity. The onset potential of MN/NGC is significantly higher than the oxidized control sample and comparable or higher than the reported values for manganese oxide‐based catalysts (Figure S18, Supporting Information).^[^ ^36^ ^]^ At 1600 rpm, the onset potential of Mn/NGC is 928 mV, comparable with the one for Pt/C (10 wt%) tested at same condition (932 mV, Figure S19a, Supporting Information). The limiting current increases linearly when the rotation speed is increased from 400 to 2400 rpm (Figure 4c). The perfectly linear Koutecky–Levich plots (Figure 4d) from 0.3 to 0.6 V (vs RHE) and the parallel fitted lines suggest first‐order reaction kinetics toward the concentration of dissolved oxygen.^[^ ^37^ ^]^ The electron transfer number calculated using slopes of the Koutecky−Levich plots is ≈4, meaning the nearly complete reduction of O_2_. These parameters demonstrate the high catalytic efficiency of the manganese nitride nanoparticles obtained. The fact that the nanoparticles are supported on the relative large carbon monolith resulted in a less direct contact between the nanoparticles and the rotating‐disk electrode.^[^ ^38^ ^]^ Therefore, the half‐wave potential of MN/NGC is lower than the one for Pt/C (Figure S19, Supporting Information). The graphitic carbon with a thickness of several layers does not affect the accessibility of electrolyte accessibility. In fact, an appropriate carbon coating has been a very effective way to improve the electrical conductivity, and maintain the core's chemical stability of the core phase in energy storage and catalysis applications.^[^ ^39^ ^]^

The pyrolysis of MOFs has become an important strategy for developing functional nanomaterials. In the present work, we have conducted a thorough mechanistic study on the pyrolysis of manganese triazolate, Mn(C_2_N_3_H_2_)_2_. New insights of the pyrolysis of MOF were gained, in terms of phase transformation, reaction pathways, and morphology evolution under different reaction conditions, which opens up new possibilities for MOF‐derived functional nanomaterials. Interestingly, the pyrolysis carried out in an oxygen‐free environment with precise process control leads to the formation of a nanoscale and air‐stable manganese nitride (Mn_2_N*_x_*), of which the synthesis has always been a challenge. The resulting Mn_2_N*_x_* nanoparticles of around 30 nm in diameter were closely embedded in nitrogen‐doped graphitic carbon layers and uniformly distributed on a carbon monolith. The existence of the graphitic carbon layers and the size match between the two species is key to prevent manganese nitride from being oxidized. In addition, the manganese nitride nanocrystallites are good electrical conductors. Thus, they show promising performance when tested for ORR electrocatalysis.

The reaction pathway of pyrolysis depends not only on the reaction conditions (e.g., whether in pure N_2_, air, or high‐vacuum environment) but also on the metal species of MOF. While the manganese triazolate gives rise to direct nitride formation, pyrolysis of the Fe and Co analogues results in the formation of Fe_3_C and cobalt metal, respectively. Such phenomenon is worthy of further study.

## Experimental Section

Chemicals used, experimental details, in situ studies, and all characterization methods can be found in the Supporting Information.

## Conflict of Interest

The authors declare no conflict of interest.

## Supporting information

Supporting Information
