# Supplementary material for: Direct Pyrolysis of a Manganese‐Triazolate Metal–Organic Framework into Air‐Stable Manganese Nitride Nanoparticles
Source: Adv Sci (Weinh). 2021 Jan 4;8(4):2003212. doi: 10.1002/advs.202003212 (PMC7887590; doi:10.1002/advs.202003212)
Supplement: Supplementary file 1 — Supporting Information [file ADVS-8-2003212-s001.pdf]

© 2020 Wiley-VCH GmbH

ADVANCED SCIENCE
Open Access

Supporting Information

for *Adv. Sci.,* DOI: 10.1002/advs.202003212

Direct Pyrolysis of a Manganese-Triazolate Metal-Organic Framework into Air-Stable Manganese Nitride Nanoparticles

Yating Hu, Changjian Li, Shibo Xi, Zeyu Deng, Ximeng Liu, Anthony K Cheetham, and John Wang**

© 2020 Wiley-VCH GmbH

Supporting Information

Direct Pyrolysis of a Manganese-Triazolate Metal-Organic Framework into Air-Stable Manganese Nitride Nanoparticles

Yating Hu, Changjian Li, Shibo Xi, Zeyu Deng, Ximeng Liu, Anthony K Cheetham, and John Wang**

Contents

Experimental Section

Figures

Table S1

DFT Calculation and Table S2

References

Experimental Section

1. Chemicals

1H-1,2,3-triazole (97%), $Mn(NO_3)_2 \cdot 4H_2O$ (≥97 %), $FeCl_2$ (99.9%), $CoCl_2$ (≥98 %), N,N-diethylformamide (DEF, 99%), N,N-dimethylformamide (DMF, 99.8%), methanol (99.9%), ethanol (99.5%), Nafion® perfluorinated resin-aqueous dispersion (10 wt.% in water) and boron nitride (98 %), Pt/C (10 wt.%) were all purchased from Sigma-Aldrich. N_2 gas cylinders (ALPHAGAZ™ 1 grade, 99.9995%) were purchased from Air Liquide Singapore.

2. Synthesis of MOFs

MET MOFs (MET-2, MET-3 and MET-4) were synthesized following Yaghi's work with some modifications.[1]

MET-2 (Manganese triazolate): 2 mmol of $Mn(NO_3)_2 \cdot 4H_2O$ was dissolved in 10 mL of DEF in a glass vial. 5 mmol of 1*H*-1,2,3-triazole was then added to the solution drop wise, and then dissolved by stirring for 30 min. The vial was capped and placed in a preheated oven at 120 ^oC for 24 h. A white solid was then formed. After cooling down to room temperature, the product was washed with DEF for once and with DMF for three times. After removing DMF solution by centrifugation, the product was immersed in methanol for 3 days and exchanging the solvent 3 times during this time. The solvent was then removed by centrifugation. The precipitation was dried in a vacuum desiccator to obtain a white and dry powder.

MET-3 (Iron triazolate): 2 mmol of $FeCl_2$ were dissolved in 12 mL of DMF by stirring in a round bottom flask. The solution was flushed with N_2 gas for 30 min to remove air. 6 mmol of 1H-1,2,3-triazole was then added to the solution drop wise. Then, under N_2 atmosphere with vigorous stirring, the mixture was heated up to 120 °C, and refluxed at that temperature for 48

h. A pink solid was then formed. After cooling down to room temperature, the product was washed with DMF for three times. After removing DMF solution by centrifugation, the product was immersed in methanol for 3 days and exchanging the solvent 3 times during this time. The solvent was then removed by centrifugation. The precipitation was then dried in a vacuum desiccator to obtain a pink and dry powder.

MET-4 (Cobalt triazolate): 2 mmol of $CoCl_2$ were dissolved in 12 mL of DMF by stirring in a round bottom flask. The solution was flushed with N_2 gas for 30 min to remove air. 6 mmol of 1*H*-1,2,3-triazole was then added to the solution drop wise. Then, under N_2 atmosphere with vigorous stirring, the mixture was heated up to 120 °C, and refluxed at that temperature for 48 h. A yellow solid was then formed. After cooling down to room temperature, the product was washed with DMF for three times. After removing DMF solution by centrifugation, the product was immersed in methanol for 3 days and exchanging the solvent 3 times during this time. The solvent was then removed by centrifugation. The precipitation was then dried in a vacuum desiccator to obtain a yellow and dry powder.

3. ***In-situ*** **Studies**

In-situ X-ray absorption near edge structure (XANES) study of the manganese triazolate's pyrolysis in purified N_2: XANES was conducted at the Singapore Synchrotron Light Source (SSLS), XAFCA beam line. 20 mg of Mn-triazolate powder were made to pallets with 80 mg of Boron Nitride. The pallet was then placed in a heating cell (about 6 cm^3 in volume) and N_2 is flushed at 100 mL/min for 1 h before the heating started. The N_2 is purified by an Oxiclear™ disposable gas purifier (in-line purifier that removes oxygen from inert carrier gases down to less than 50 ppb). The cell was heated to 525 ^oC (the temperature where the XANES signal stabilized) at 5 ^oC/min ramp rate, and then held at that

temperature. During the heating, N_2 is flowing at 100 mL/min. XANES is continuously scanned under transmission mode. Each scan takes 6 min.

Thermogravimetric analysis–mass spectrometry (TG-MS) test of the manganese triazolate: TG-MS was conducted on a Mettle Toledo thermogravimetric analyzer, coupled with a Pifzer mass spectrometry, which is capable of qualitative analysis of evolved gases during thermal decomposition. Range of mass detected: 1-100 AMU (atomic mass unit). About 6-10 mg of manganese triazolate was put in an aluminum pan inside the chamber. Then, the sample was heated to 600 ^{o}C at the ramp rate of 5 ^{o}C/min in air or N_2, flowing at 100 mL/min. When done in N_2, N_2 is flushed for 30 min before the heating started. Signals of below AMUs were collected while only the detected ones are shown in the results: 2 (H_2), 16 (CH_4), 17 (NH_3), 28 (N_2), 30 (NO), 44 (CO_2), 46 (NO_2), 55(Mn), 69 ($C_2H_3N_3$).

In-situ scanning transmission electron microscopy (STEM) study of the manganese triazolate's pyrolysis in the STEM chamber: In-situ STEM study was conducted on a JEOL ARM200f microscope at an acceleration voltage of 200 kV, using the *in-situ* heating and biasing holder (DENSsolutions) with heating MEMS chips with Si_3N_4 membrane. Powder of manganese triazolate was well dispersed in ethanol by ultrasonication, and then dropped onto the chip. The temperature accuracy is within 5%. The base vacuum is about 1e-7 mbar. No oxygen trap is installed.

4. Pyrolysis/annealing of the MOFs

About 60-100 mg of the as-synthesized MOF powder was placed in an alumina crucible. The crucible was then placed in a tube furnace connected with mechanical and turbo pumps. At the N_2 inlet, an oxygen/moisture trap: Oxiclear™ disposable gas purifier (in-line purifier that removes oxygen from inert carrier gases down to less than 50 ppb) was connected. A vacuum level of ~5E-4 mbar was obtained before filling with N_2 gas to atmospheric pressure. The above degassing/N_2-filling cycle was

repeated for 3 times. Then, the MOF went through heat-treatment with N_2 flowing at 300 mL/min. Ramp rate of 5 °C/min was used for all processes while various temperature and durations were used. The product was retrieved upon cooling down to room temperature. The table below shows the sample nomenclature based on different heat-treatment conditions.

	**Annealing temperature (^oC)**	**Annealing time (h)**
**MN/NGC**	525	4
**MN/NGC (6h)**	525	6
**MET2-N600**	600	4
**MET2-N650**	650	4

5. <u>Characterizations</u>

The samples derived from the various pyrolysis/annealing processes were then analyzed using STEM, transmission electron microscopy (TEM), X-ray diffraction (XRD), energy dispersive X-ray analysis (EDX), CHNS analyzer, inductively coupled plasma-optical emission spectrometer (ICP-OES). XRD patterns were obtained using a Bruker AXS X-ray powder diffractometer (D8 Advance, Cu Kα, λ = 0.15418 nm). Elemental analysis was conducted by using the CHNS (Carbon, Hydrogen, Nitrogen and Sulphur) analyzer (Elementar vario MICRO cube) for carbon, nitrogen and hydrogen contents and ICP-OES (Perkin Elmer Optima 5300DV) for manganese contents. Field-emission scanning electron microscopy (SEM) were conducted using a SUPRA 40 ZEISS. TEM imaging were conducted using a JEOL JEM-2010 microscope under an acceleration voltage of 200 kV. STEM imaging, EDX mapping and electron energy loss spectroscopy (EELS) study were conducted using a JEOL

ARM200f microscope and an Oxford X-max 100TLE SDD energy dispersive X-ray spectroscopy (EDS) under an acceleration voltage of 200 kV. Specific surface area and pore size distribution were calculated from N_2 adsorption/desorption measurement (Micromeritics 3Flex).

Thermal gravimetric analysis (TGA) of the MOF was done using a TA Instruments Q500. Helium ion microscopy (HIM) images were taken using ZEISS NanoFab system. He ion accelerating voltage is 30 kV and beam current is 1 pA.

6. <u>Electrochemical measurements and calculations.</u>

Oxygen reduction reaction (ORR) catalytic activity and electrical impedance spectroscopy (EIS) of the samples were studied in a standard three-electrode system in O_2-saturated 0.1 M KOH solution (pH=12.77). A saturated calomel electrode (SCE) and a graphite rod were used as the reference electrode and counter electrode, respectively. ORR catalytic activity was tested using a WaveDriver 200 EIS Bipotentiostat/Galvanostat and a WaveVortex 10 Electrode Rotator from Pine Instruments. EIS was tested using a Solartron System 1400A.

Preparation of working electrodes: 5 mg of each active materials and 10 μl of Nafion solution (10 wt.% in water) were uniformly dispersed in a mixture of 490 μl of H_2O and 500 μl of ethanol with the assistance of ultrasonication for 1-2 h until no visible solid in the dispersion. 12 μl of the dispersion was then dropped onto a glassy-carbon rotating-disk electrode (disk area: 0.196 cm^2) from Pine Instruments and dried in air. Loading of active material was 0.3 mg/cm^2.

ORR polarization curves were collected at various rotation rates ranging from 400 to 2400 rpm and the curve at 1600 rpm was used to calculate the onset potential. EIS was tested from 10^6 to 1 Hz.

The onset potential is the potential at where the current reaches 5% of limiting current, which is defined based on the methods described in Figure S17b.

The electron transfer number *n* is calculated based on the slopes of the Koutecky-Levich plots (Figure S16) and Koutecky-Levich equation.[2]

7. Statistical Analysis

Fourier transform of the extended X-ray absorption fine structure (EXAFS) spectra were in the k-range of 2.0 to 9.0. Sample size for the particle size distribution in Fig. S10 is 60 (more details could be found in Fig. S10's caption). Background noises are subtracted for XRD curves. Details for density function theory (DFT) calculation related to total energy can be found in "DFT Calculation" section, which is listed at the end of supporting information.

Figures:

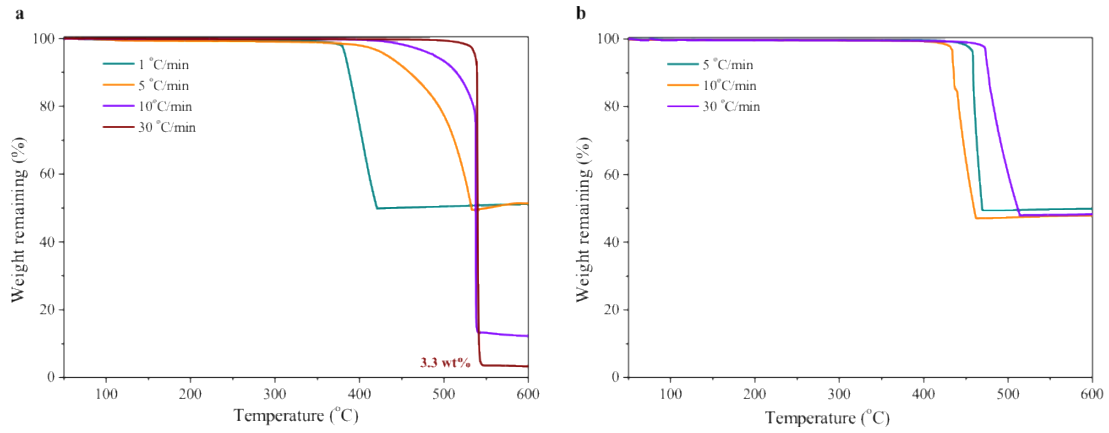

Figure S1. TGA results of manganese triazolate in (a) N_2 and (b) air at different ramp rates.

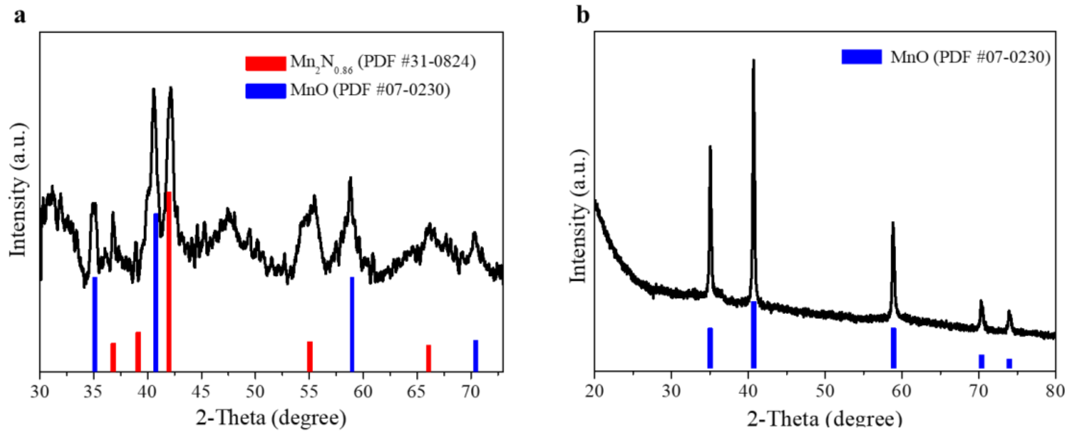

Figure S2. XRD patterns of manganese triazolate pyrolyzed/annealed in (a) N_2 but without using oxygen trap and (b) air.

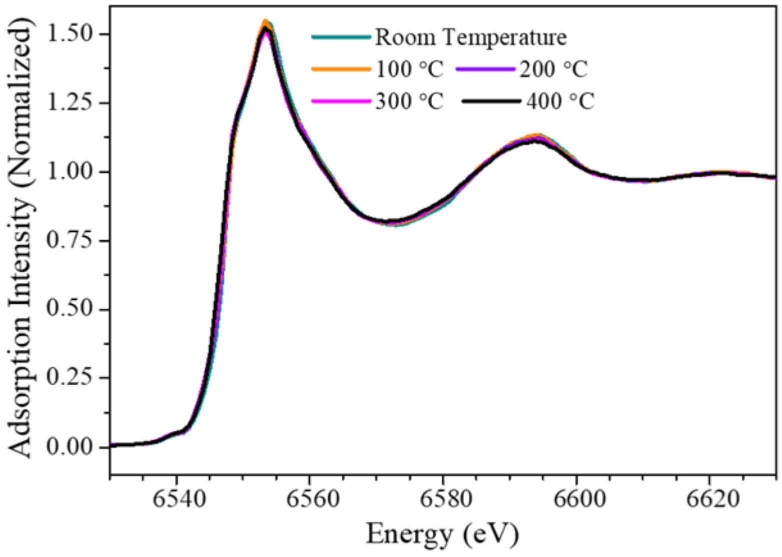

Figure S3. *In-situ* XANES spectra collected when manganese triazolate is heated from room temperature to 400 °C, in purified N_2.

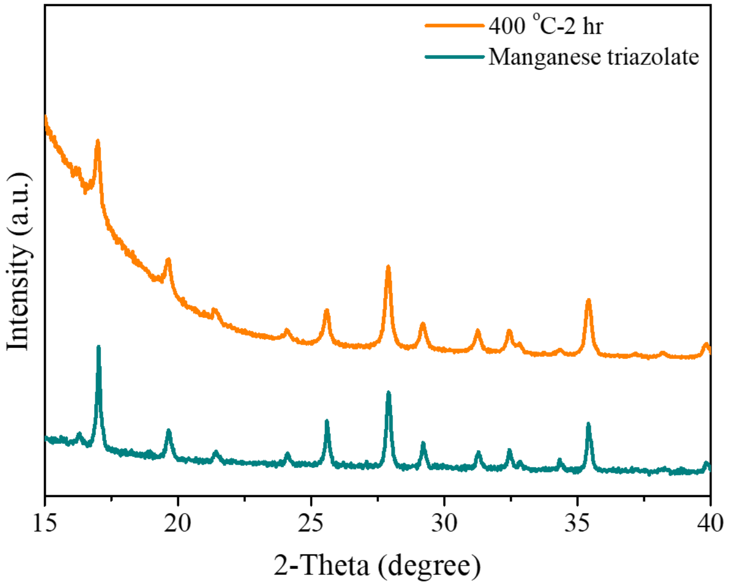

Figure S4. XRD patterns of the as-synthesized manganese triazolate (MOF MET-2) and after heat-treatment at 400 °C for 2 hours, in N_2. The rather identical peak positions before and after the heat-treatment proved that the manganese triazolate is thermally stable up to 400 °C.

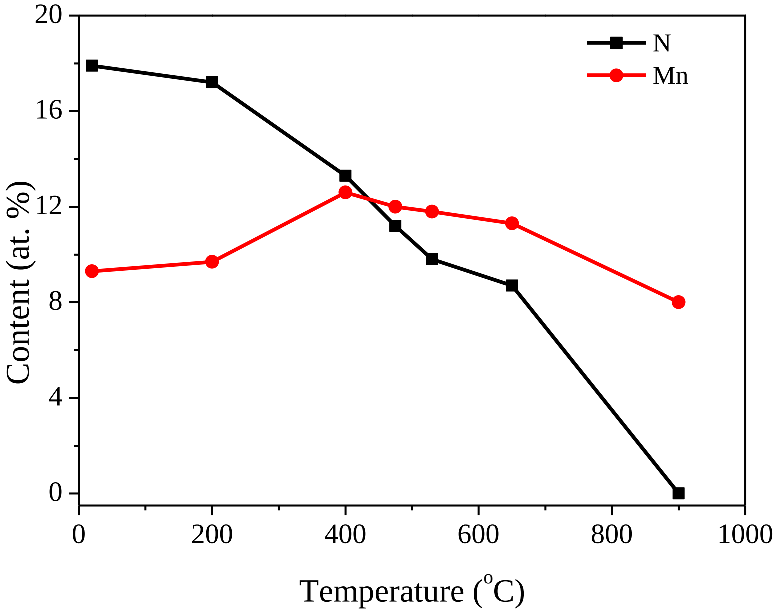

Figure S5. Element content of N and Mn at different temperatures during the *in-situ* STEM imaging, calculated from the electron energy loss spectroscopy analysis.

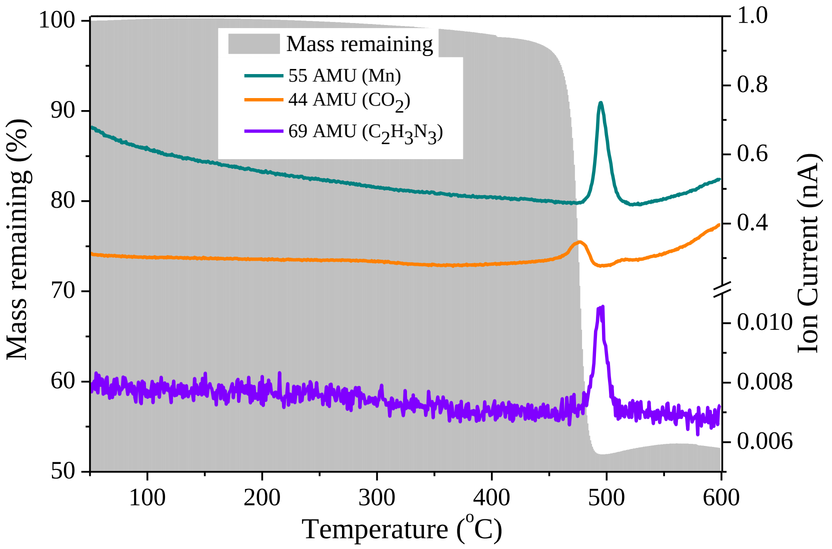

Figure S6. TG-MS results during the pyrolysis of manganese triazolate in air at a ramp rate of 5 °C /min.

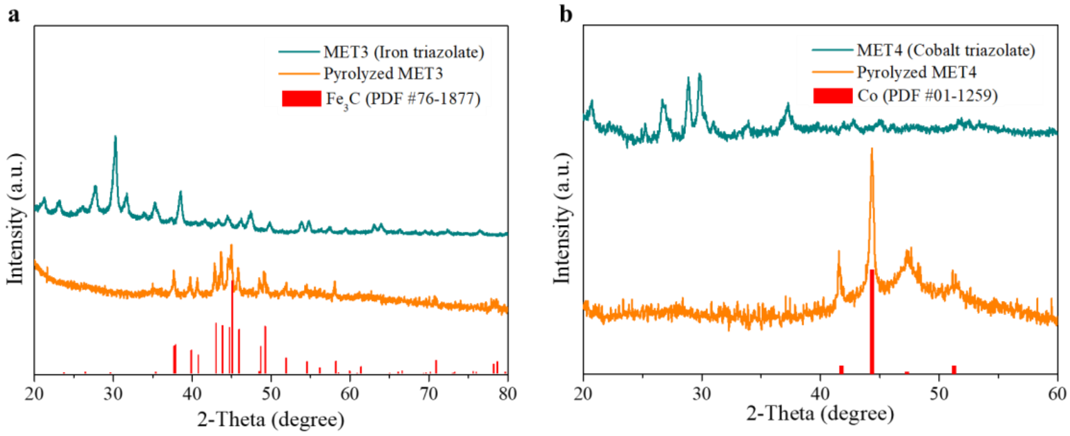

Figure S7. XRD patterns of (a) Fe and (b) Co-based METs, and iron carbide or cobalt metal obtained after pyrolysis at 525 °C for 4h, in purified N_2.

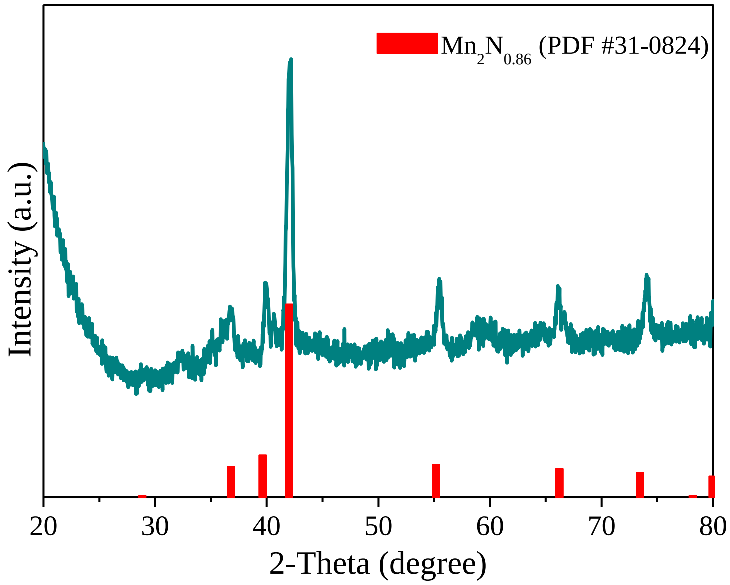

Figure S8. XRD pattern of the pyrolyzed product of manganese triazolate when switching N_2 to Ar (525 °C for 4 h).

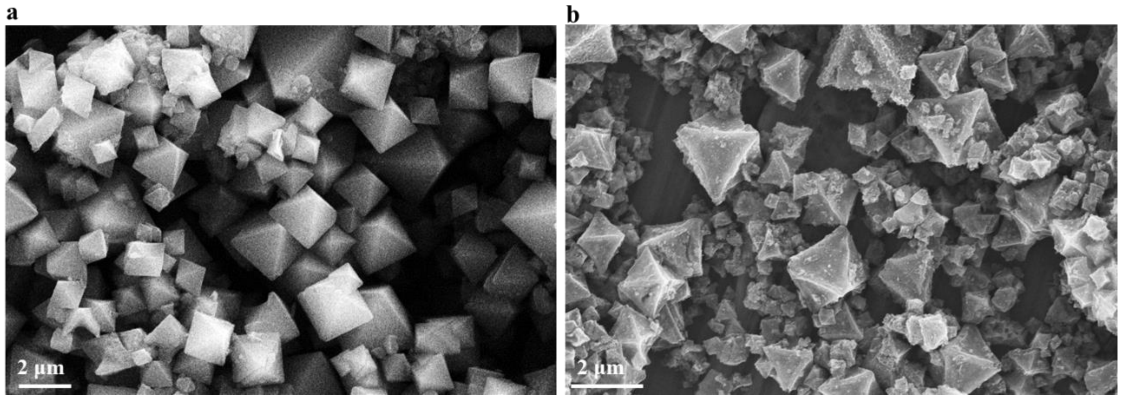

Figure S9. SEM images of the (a) manganese triazolate and (b) MN/NGC.

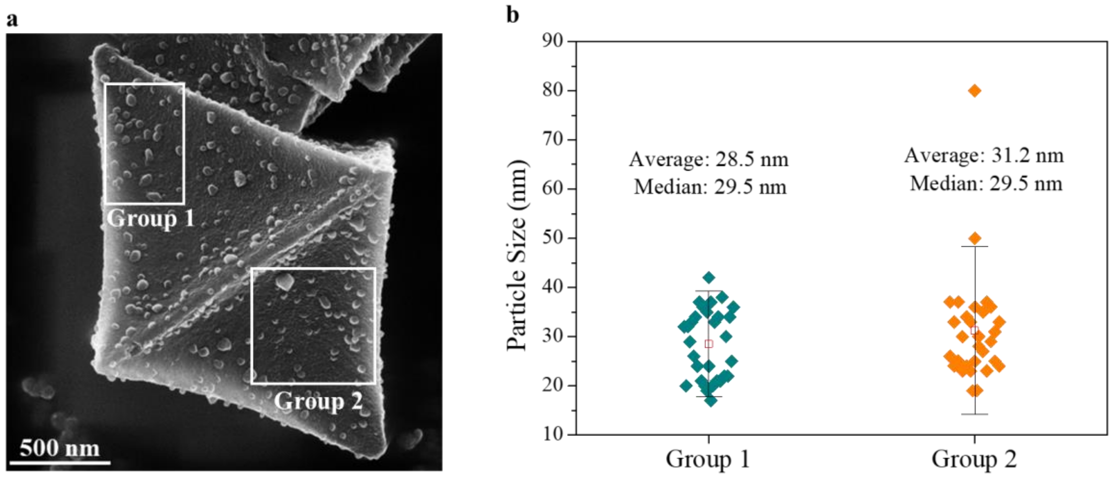

Figure S10. Particle size distribution of the manganese nitride nanoparticles of sample MN/NGC. Two groups consisting of around 30 nanoparticles were selected randomly from the HIM image of the MN/NGC. Over 90% nanoparticles in both groups are in the size of 20-40 nm, with average size of 28.5 and 31.2 for Group 1 and 2, respectively. Thus, it is concluded that the particle size of the manganese nitride nanoparticles is 30±10 nm.

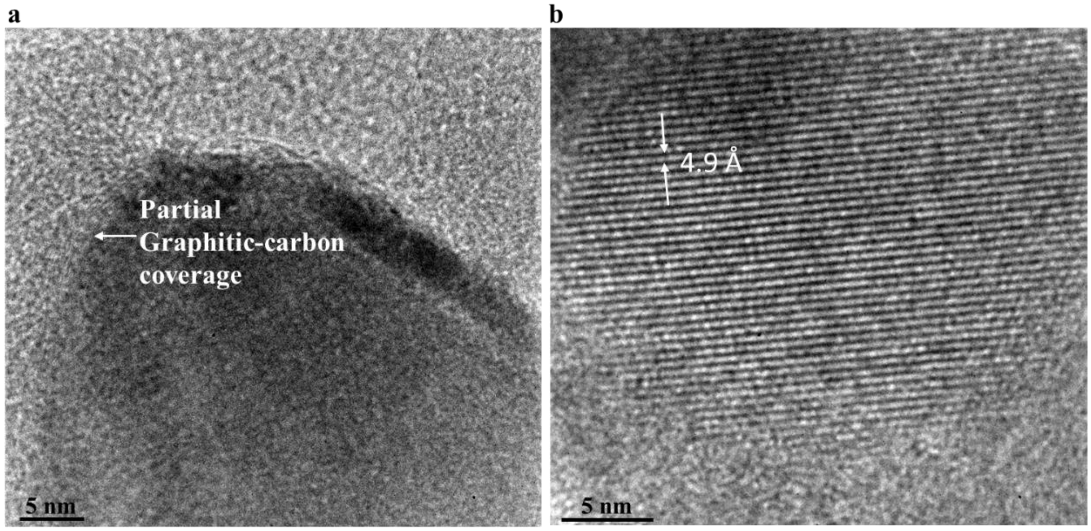

Figure S11. TEM images of the MN/NGC (6h). Lattice fringe of 4.9 Å in (b) corresponds to the (101) plane of hausmannite Mn_3O_4 (PDF #18-0803).

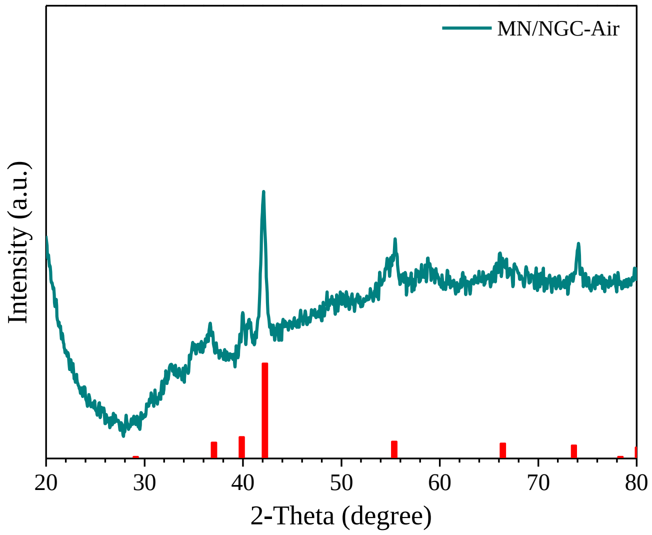

Figure S12. XRD of the MN/NGC after being stored in air for 4 weeks (MN/NGC-Air).

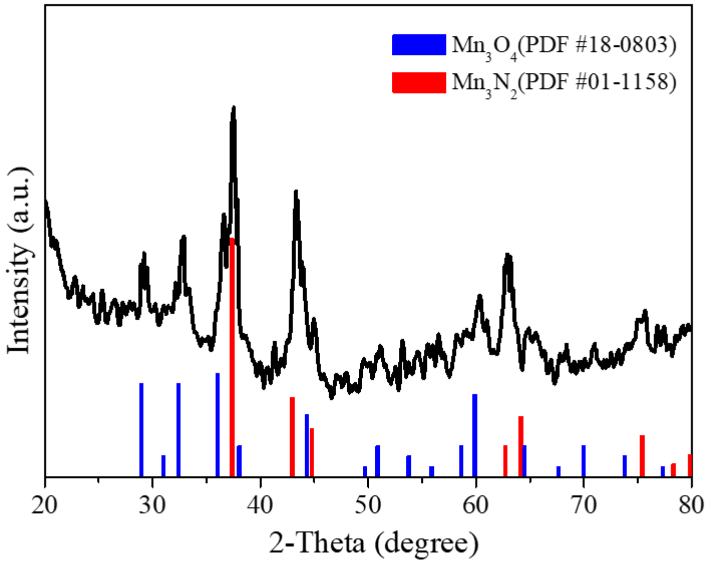

Figure S13. XRD result of bulk Mn_2N_3 synthesized based on a previously reported method and it was immediately oxidized into Mn_3O_4.[3]

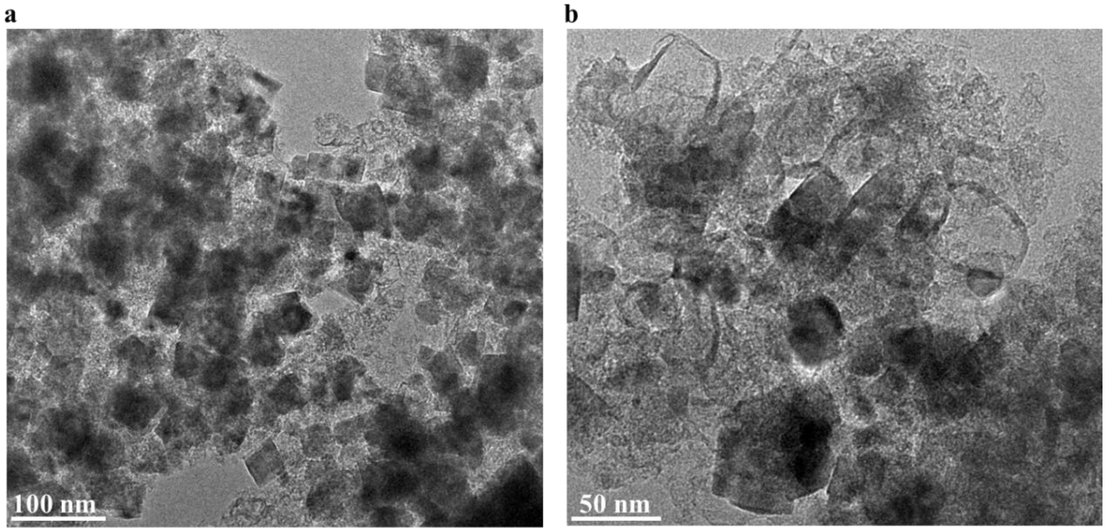

Figure 14. TEM images of (a) MET2-N600 and (b) MET2-N650. Graphitic carbon layers were not retained and the cubic particles are Mn_3O_4 nanocrystals.

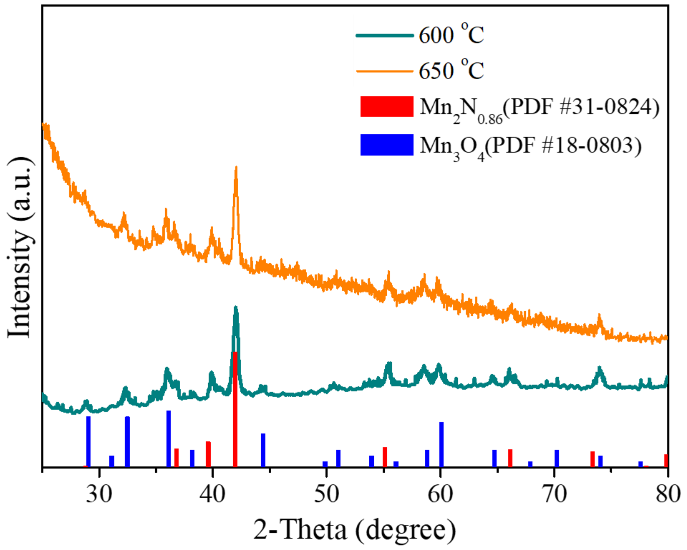

Figure 15. XRD results of MET2-N600 and MET2-N650.

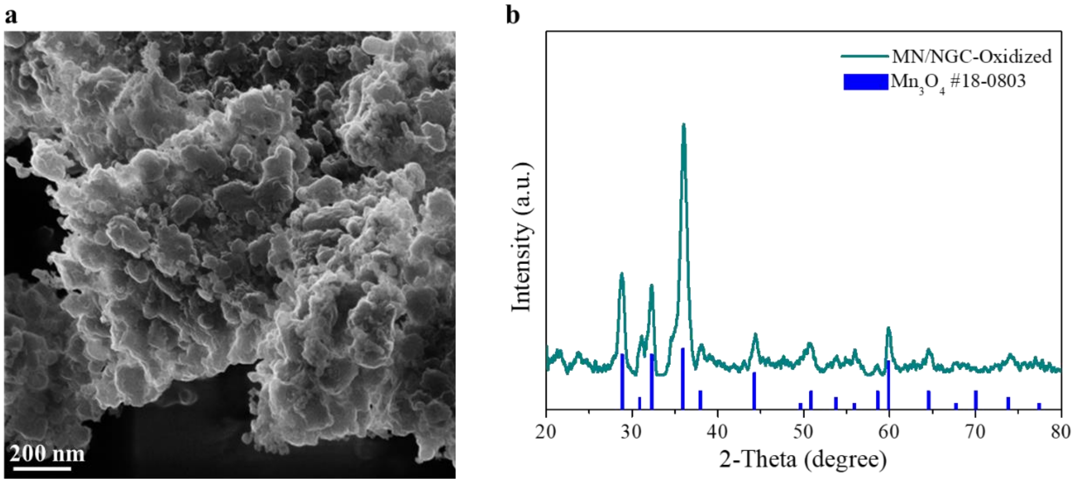

Figure S16. HIM image and XRD pattern of MN/NG-oxidized.

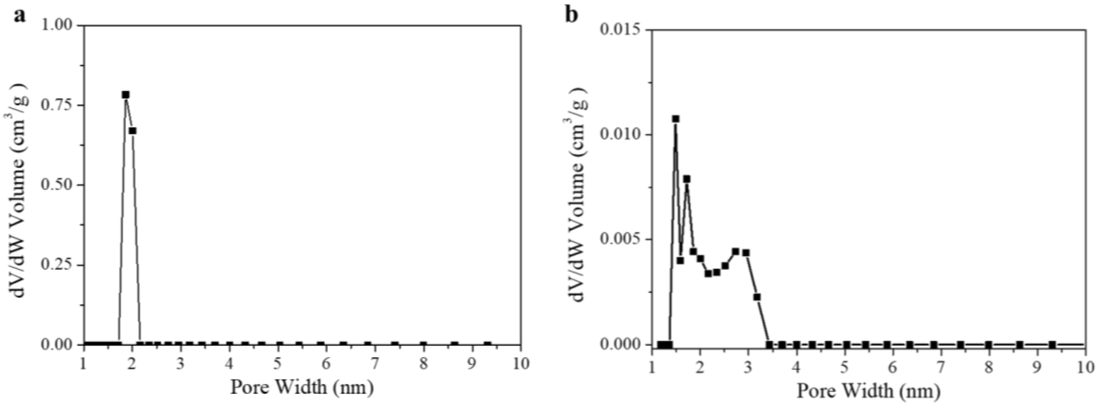

Figure S17. Pore size distributions calculated from the N_2-adsorption/desorption test using the density function theory (DFT) model: (a) manganese triazolate (sharp peak at 1.9 nm), and (b) MN/NGC (a range of 1.5 to 3.0 nm). As a result of the MOF collapse, the Brunauer-Emmett-Teller surface area calculated from N_2 adsorption was reduced from 908 to 113 m^2/g (from the as-synthesized manganese triazolate to the MN/NGC).

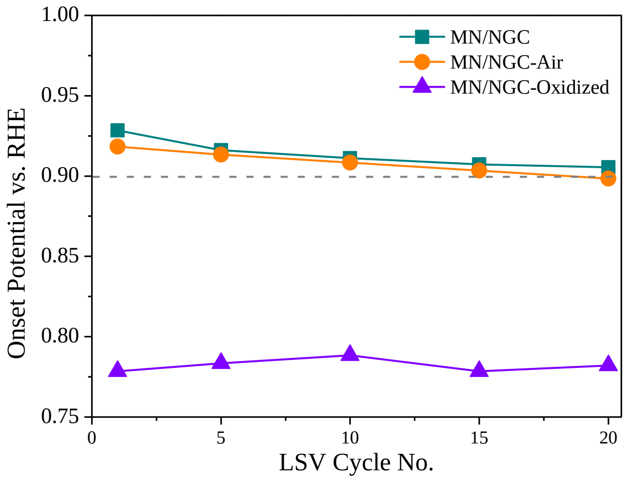

Figure S18. Onset potential at different cycles for various samples tested at the rotation speed of 1600 rpm.

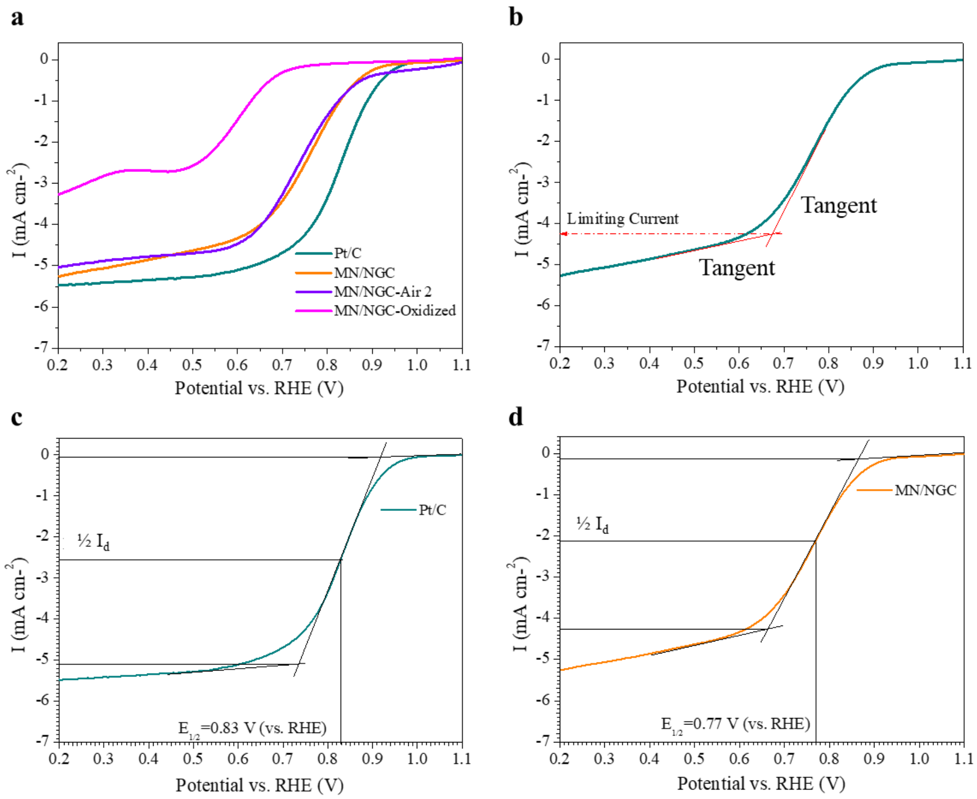

Figure S19. (a) LSV curves of Pt/C and all samples tested for ORR, at 1600 rpm; (b) the definition of limiting current: intersection of the two tagent lines coresponds to the limiting current; Diffusion-limiting current I_d and half-wave potential $E_{1/2}$ of (c) Pt/C and (d) Mn/NGC.

Table S1. Chemical compositions obtained from CHNS and ICP-OES tests.

	C	N	Mn	O (Deduced)
**Manganese Triazolate**	26.7	39.1	6.6	1.3
**MN/NGC**	39.2	22.8	22.5	5.5
**MN/NGC-Air**	37.2	22.7	22.0	7.3
**MN/NGC (6h)**	37.4	21.5	18.4	12.9
**MET2-N600**	34.2	11.5	18.2	19.8
**MN/NGC-Oxidized**	29.6	14.8	20.1	24.8

Samples are dissolved in Aqua Regia for 30 min before being tested by CHNS element analyzer and ICP-OES. As Aqua Regia is oxidizing, the deduced O's at.% might be higher than the actual value. For example, pure manganese triazolate does not contain any O, the tested value 1.3 at% could well be due to a minor oxidation from the Aqua-regia treatment.

DFT Calculation

Computational Methods: The spin polarized DFT calculations were performed using the Vienna ab initio Simulation Package[4, 5] with the Projected Augmented Wave pseudopotential[6, 7] and the following electrons were treated explicitly: H $1s,^1$ C $2s^22p,^2$ N $2s^22p^3$ and Mn $4s^23d^5$. A plane wave kinetic energy cutoff of 520 eV and a Γ-centered Monkhorst-Pack[8] k-point mesh with a line density of 25 were used. Van der Waals forces were included using the Grimme's scheme (DFT+D3).[9] Geometry optimizations were performed until the interatomic forces becomes smaller than 0.01 eV/Å. The calculations of the phases containing Mn started from the ferromagnetic case in which all of the spins were aligned to the same direction. The decomposition enthalpy was calculated using:

$$H_d = 0.5E[Mn_2N]+3E[C(graphite)]+E[CH_4(g)]+2.75E[N_2(g)]-E[Mn(C_2H_2N_3)_2]$$

where E is the DFT total energy. The DFT total energy of the CH_4 and N_2 in gas phase were calculated by putting a single molecule in the center of a 20 x 20 x 20 Å^3 box. Mn_2N_x(x=0.86 based on XRD results) is simplified into Mn_2N for the benefit of calculation. The decomposition Gibbs free energies at different temperature T were calculated as

$$G_d(T) = H_d-T(S[CH_4,T]+2.75S[N_2,T])$$

where S is the entropy of the CH_4 and N_2 in gas phase, as shown in Supplementary Table S2.1, calculated from the data obtained from the NIST table as shown in Supplementary Table S2.2.

Table S2. Calculated decomposition Gibbs free energies (G_d) at 300, 400 and 500 K based on the overall reaction proposed in Fig. 1e. Units are in kJ/mol per $Mn(C_2H_2N_3)_2$. At 0 K, G_d is the same as the decomposition enthalpy (H_d).

T (K)	0	300	400	500
G_d	-686.32	-897.64	-980.40	-1066.19

Table S2.1. Calculated entropy (unit: J mol^{-1} K^{-1}) at 300, 400 and 500 K using $S = A \ln T + B T + C T^2/2 + D T^3/3 - E / (2T^2) + G$, where A, B, C, D, E, G are shown in Supplementary Table 2.2.

T (K)	CH_4	N_2
300	0.19	0.19
400	0.20	0.20
500	0.21	0.20

Table S2.2. Constants taken from NIST webbook[10] used for entropy calculations.

Formula	A	B	C	D	E	G
CH_4	-0.70	108.48	-42.52	5.86	0.68	158.72
N_2	28.99	1.85	-9.65	16.64	0.00	226.42

References

[1] F. Gándara, F. J. Uribe-Romo, D. K. Britt, H. Furukawa, L. Lei, R. Cheng, X. Duan, M. O'Keeffe, O. M. Yaghi, *Chem. Eur*, **2012,** 18, 10595.

[2] Y. Lu, Y. Jiang, X. Gao, X. Wang, W. Chen, *J. Am. Chem. Soc.*, **2014,** 136, 11687.

[3] A. Leineweber, R. Niewa, H. Jacobs, W. Kockelmann, *J. Mater. Chem.*, **2000,** 10, 2827.

[4] G. Kresse, J. Furthmüller, *Physical Review B*, **1996,** 54, 11169.

[5] G. Kresse, J. Furthmüller, *Computational Materials Science*, **1996,** 6, 15.

[6] G. Kresse, D. Joubert, *Physical Review B*, **1999,** 59, 1758.

[7] P. E. Blöchl, *Physical Review B*, **1994,** 50, 17953.

[8] H. J. Monkhorst, J. D. Pack, *Physical Review B*, **1976,** 13, 5188.

[9] S. Grimme, J. Antony, S. Ehrlich, H. Krieg, *The Journal of Chemical Physics*, **2010,** 132, 154104.

[10] P. J. Linstrom, W. G. Mallard, *NIST Chemistry WebBook*. 2020, National Institute of Standards and Technology: Gaithersburg MD, 20899.
